# Dietary protein and fat intake in relation to risk of colorectal adenoma in Korean

**DOI:** 10.1097/MD.0000000000005453

**Published:** 2016-12-09

**Authors:** Sun Young Yang, Young Sun Kim, Jung Eun Lee, Jueun Seol, Ji Hyun Song, Goh Eun Chung, Jeong Yoon Yim, Sun Hee Lim, Joo Sung Kim

**Affiliations:** a Department of Internal Medicine, Healthcare System Gangnam Center, Seoul National University Hospital; b Department of Food and Nutrition, Seoul National University; c Department of Food and Nutrition, Sookmyung Women's University; d Department of Internal Medicine, Liver Research Institute, Seoul National University College of Medicine, Seoul, Republic of Korea.

**Keywords:** colorectal adenoma, dietary, fat, macronutrient, protein, risk factor

## Abstract

Consumption of red meat and alcohol are known risk factors for colorectal cancer, but associations for dietary fat remain unclear. We investigated the associations of dietary fat, protein, and energy intake with prevalence of colorectal adenoma.

We performed a prospective cross-sectional study on asymptomatic persons who underwent a screening colonoscopy at a single center during a routine health check-up from May to December 2011. Dietary data were obtained via a validated Food Frequency Questionnaire (FFQ), assisted by a registered dietician. We also obtained information on alcohol consumption and smoking status, and measured metabolic syndrome markers including abdominal circumference, blood pressure, fasting glucose, serum triglyceride and high-density lipoprotein cholesterol. We calculated odds ratio (OR) and 95% confidence interval (CI) to evaluate the associations using the polytomous logistic regression models. As a secondary analysis, we also conducted a matched analysis, matched by age and sex (557 cases and 557 non-cases).

The study sample included 557 cases (406 males and 151 females) with histopathologically confirmed colorectal adenoma, and 1157 controls (650 males and 507 females). The proportion of advanced adenoma was 28.1% of men and 18.5% of female, respectively. Although vegetable protein intake was inversely associated with the prevalence of colorectal adenoma, further adjustment for potential confounding factors attenuated the association, resulting in no significant associations. There were no significant associations between dietary fat intake and colorectal adenoma in energy-adjusted models. For vegetable protein in women, the OR for the comparison of those in the highest tertile with those in the lowest tertile was 0.47 (95% CI 0.25–0.91, *P* for trend = 0.07) after adjustment for total energy intake. However, after controlling for metabolic syndrome markers, body mass index, smoking status, alcohol consumption, and family history of colorectal adenoma, which were all significantly high in the colorectal adenoma patients group, the association became attenuated (OR 0.54, 95% CI 0.27–1.11, *P* for trend = 0.13).

In conclusion, we did not observe the significant associations for intakes of total energy, total, animal and vegetable fats, and total, animal and vegetable proteins in relation to colorectal adenoma prevalence.

## 1 Introduction

Colorectal cancer has been 1 of the most common cancers in Western countries, and the incidence rate has recently been increasing in Asian countries. In Korea, the incidence rates of colorectal cancer have continued to increase in both sexes, resulting in becoming the second most common cancer in males and the third most common cancer in females.^[1]^ It may be partly due to shift to Western lifestyle such as diet, physical activity, and increase in the obese population. In general, physical activity, low total energy intake, low red and processed meat consumption, and limited alcohol drinking were known to give beneficial effect for cancer prevention.^[2]^ Diet has long been regarded as the most important lifestyle risk factor for colorectal cancer. However, role of many dietary factors in colon carcinogenesis remains unresolved.

In animal and in vitro studies which investigated the effect of dietary proteins on colorectal cancer, high protein diet led to DNA damage of colonocytes, decreasing colonic mucosal thickness, and reduction of the height of colonocyte brush-border membrane.^[3–5]^ On the contrary, another study showed that curcumin, the active ingredient of the Indian spice turmeric, had chemopreventive effect on high protein diet-associated colorectal cancer in rat.^[6]^

Inverse association between the intake of vegetables, fruits, or fish oil and risk of colorectal cancer has been suggested in several meta-analyses,^[7,8]^ whereas meat consumption was significantly associated with an increased risk of colorectal cancer.^[9]^ In animal studies, dietary fat induced the secretion of bile acids, which result in injury and regression of colonic mucosal epithelium, finally increasing the risk of colorectal cancer.^[10,11]^ However, the effect of dietary fat on colorectal cancer has been inconsistent in epidemiological studies.^[12,13]^ A recent meta-analysis suggested that dietary fat may not be associated with the increased risk of colorectal cancer.^[14]^ But, most of the included studies were performed in Europe and America, so the results are difficult to extrapolate to the Asian population. Furthermore, there were only a few prospective studies to examine the association of dietary intake and colorectal cancer.

Colorectal adenomas are considered precursors to colorectal cancer through adenoma-carcinoma sequence.^[15]^ The identification of modifiable risk factors for colorectal adenoma contributes to prevent colorectal cancer from progressing. Studies that examined dietary risk factors for colorectal adenoma reported that fat and red meat intake increased the risk for colorectal adenoma, whereas fiber, fruit, and vegetable intake decreased the risk.^[16,17]^ However, findings with regard to dietary risk factors are still controversial. According to 1 study, whereas the intake of fresh vegetables or fiber of the colorectal adenoma group was not particularly higher than that of the control group, it still significantly decreased the risk of progression of advanced adenoma in colorectal cancer in the carcinoma-adenoma sequence.^[18]^ Meanwhile, a recent meta-analysis reported that vegetable intake was not associated with the incidence of colorectal adenoma, whereas fruit intake was found to have protective effects against colorectal adenoma.^[19]^ As such, various research findings regarding dietary risk factors of colorectal adenoma have been reported. In addition, although high-fat and high-protein diets are known as dietary risk factors of colorectal adenoma, clinical studies have failed to provide sufficient evidence supporting this. Moreover, as in the case of colorectal cancer, Western research findings on the dietary risk factors of colorectal adenoma in Asians whose lifestyle and diet differ from those of Westerners are difficult to apply. Furthermore, as with colorectal cancer, it would be incorrect to simply apply Western research data on risk factors of colorectal adenoma.

In this regard, this study aimed to examine the associations between the intakes of dietary protein and fat and prevalence of colorectal adenoma in Koreans by comparing the macronutrient intakes of Korean patients with colorectal adenoma and advanced adenoma with those of a normal control group.

## 2 Methods

### 2.1 Study population

We performed a prospective cross-sectional study on asymptomatic persons, who underwent a colonoscopy at Seoul National University Hospital Healthcare System Gangnam Center during a routine health check-up from May to December 2011. Colonoscopy was conducted after nutritional surveys on the same day. And thereafter, colorectal adenoma patients and adenoma-free control group was setup after colonoscopy.

We included a total of 557 patients and 1157 controls, aged 30 to 76 years. Participants were asked about socio-demographic status, alcohol consumption, smoking status, and family history of colorectal cancer. We also measured metabolic syndrome marker including abdominal circumference, blood pressure (BP), fasting glucose, serum triglyceride and high-density lipoprotein (HDL) cholesterol. Polyps were verified as adenomatous, hyperplastic, or inflammatory through pathological examination by colonoscopies. If participants had a diagnosis of colorectal adenoma, we included as cases.

Patients were excluded as follows: incomplete study due to poor bowel preparation and cecal intubation failure, and past history of colorectal adenoma, colorectal cancer, inflammatory bowel disease, intestinal tuberculosis, and bowel resection. We also excluded the patients with diagnosis of colorectal cancer (n = 7) or unreasonable energy intake (beyond mean of logarithm of energy ± SDs) (n = 6).

The study protocol was approved by the Institutional Review Board of Seoul National University Hospital, and written informed consents were obtained from all participants.

### 2.2 Dietary intake

Dietary intake was assessed before colonoscopy at the same day using a validated Food Frequency Questionnaire (FFQ),^[20]^ assisted by a registered dietician. Participants were asked to estimate their usual frequency of consumption of various foods and typical portion sizes for the year preceding the interview date. Each food item had 9 options for frequency (ranging from “never or less than once per month” to “3 times per day”) and 3 options for portion size. For these analyses, we examined total energy intake and the following macronutrients: total protein; animal protein; vegetable protein; total fat; animal fat; and vegetable fat.

### 2.3 Assessment of risk factors

Metabolic syndrome was diagnosed when 3 or more of the following 5 criteria were present according to the modified Adult Treatment Panel III (ATP III), as adopted by the updating guidelines of the National Cholesterol Education Program (NCEP-ATP III)^[21]^: central obesity (waist circumference >90 cm [men] or >80 cm [women]); a triglyceride level ≥150 mg/dL; HDL cholesterol<40 mg/dL (men) or <50 mg/dL (women); fasting plasma glucose ≥100 mg/dL or treatment for diabetes; systolic BP ≥130 mm Hg or diastolic BP ≥85 mm Hg or treatment for hypertension.

With regard to smoking, current smokers were defined as those who had been smoking at least 1 cigarette per day during the previous 12 months.

An advanced adenoma was specified a presence of adenoma with villous component (at least 25% villous structure), with high-grade dysplasia, ≥10 mm in estimated diameter and/or presence of 3 or more synchronous adenomas.

### 2.4 Statistical analysis

We calculated the means and SDs, and compare baseline characteristics between cases and non-cases using a *t* test for continuous variables. For categorical variables, we calculated the proportion of characteristics and conducted a chi-square test. Animal fat, vegetable fat, animal protein, and vegetable protein intake was adjusted for energy by residual methods.^[22]^ We calculated odds ratio (OR) and 95% confidence interval (CI) to evaluate the associations using the polytomous logistic regression models. As a secondary analysis, we also conducted a matched analysis, matched by age and sex (557 cases and 557 non-cases). For test for trends, the median value of each tertile in control group was included in the models as a continuous variable. We adjusted for age (years, continuous), sex (men or women), total energy intake (kcal/d, continuous), waist circumstance (cm, continuous), smoking status (never, past smoker, current smoker), alcohol intake (g/d, continuous), body mass index (BMI, kg/m^2^, continuous), HDL cholesterol (mg/dL, continuous), fasting glucose (mg/dL, continuous), and family history of colorectal cancer (yes or no). All analyses were performed using SAS 9.3 (SAS Institute Inc., Cary, NC). All statistical tests were 2-sided, and *P* values <0.05 were considered statistically significant.

## 3 Results

### 3.1 Baseline characteristics of subjects

Demographic and clinical characteristics of the study participants, stratified by sex and case-control status, are shown in Table 1. The study sample included 557 cases (406 males and 151 females) with histopathologically confirmed colorectal adenoma (38.4% male and 22.9% female, respectively) and 1157 controls (650 males and 507 females). The proportion of advanced adenoma was 28.1% of men and 18.5% of women, respectively. Colorectal adenoma cases were slightly older and had a higher triglyceride and fasting glucose than controls in both males and females. Female patients had a higher BMI and lower HDL cholesterol, and drank more alcohol than controls. Mean macronutrient intakes and total average energy intake of cases and controls were not significantly different in both sexes (Table 1).

**Table 1:**
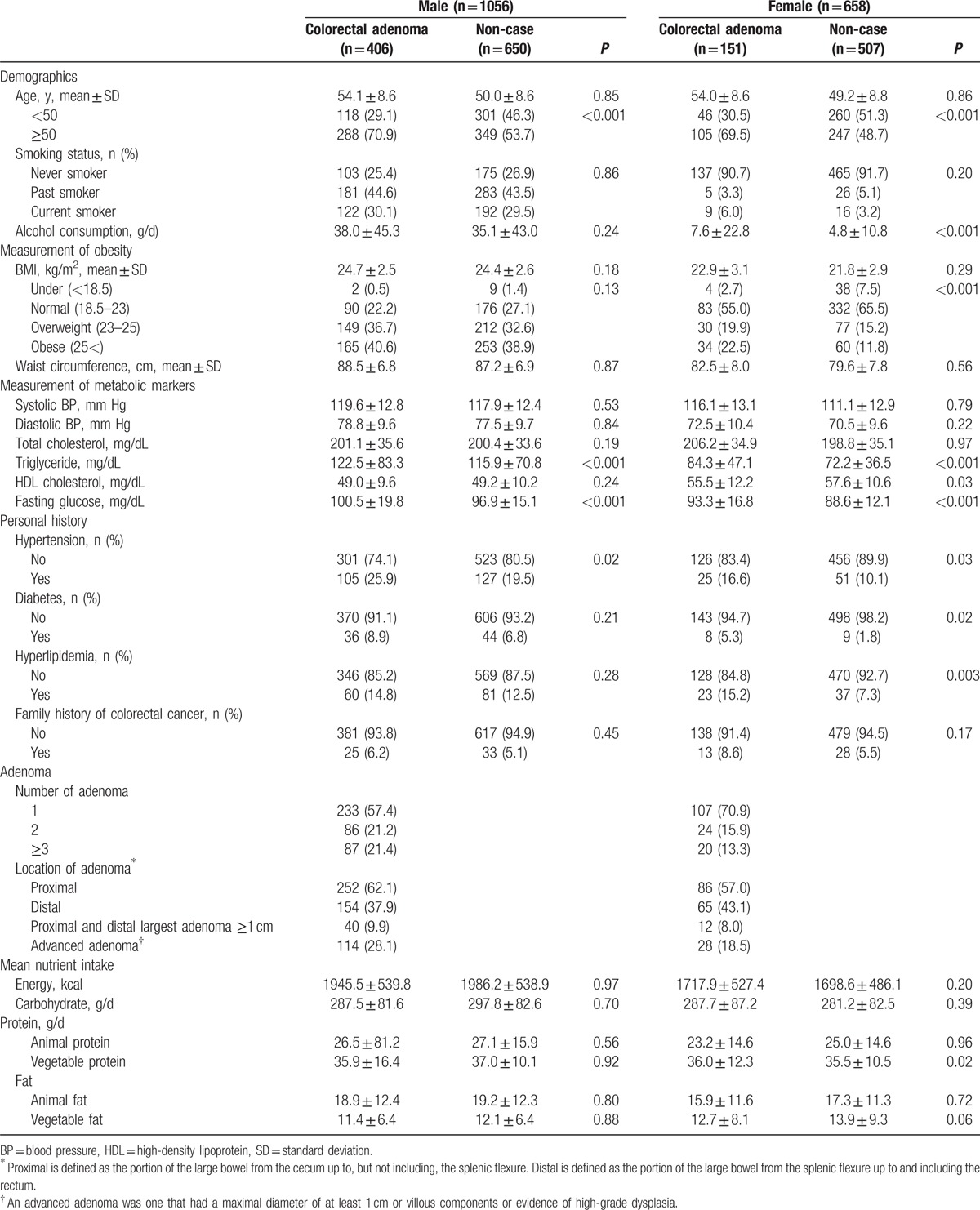
Baseline characteristics of patients and controls.

### 3.2 Standard multivariate model and substitution model

Although vegetable protein intake was inversely associated with the prevalence of colorectal adenoma, further adjustment for potential confounding factors attenuated the association, resulting in no significant associations (Table 2). There were no significant associations between dietary fat intake and colorectal adenoma in energy-adjusted models (Table 3).

**Table 2:**
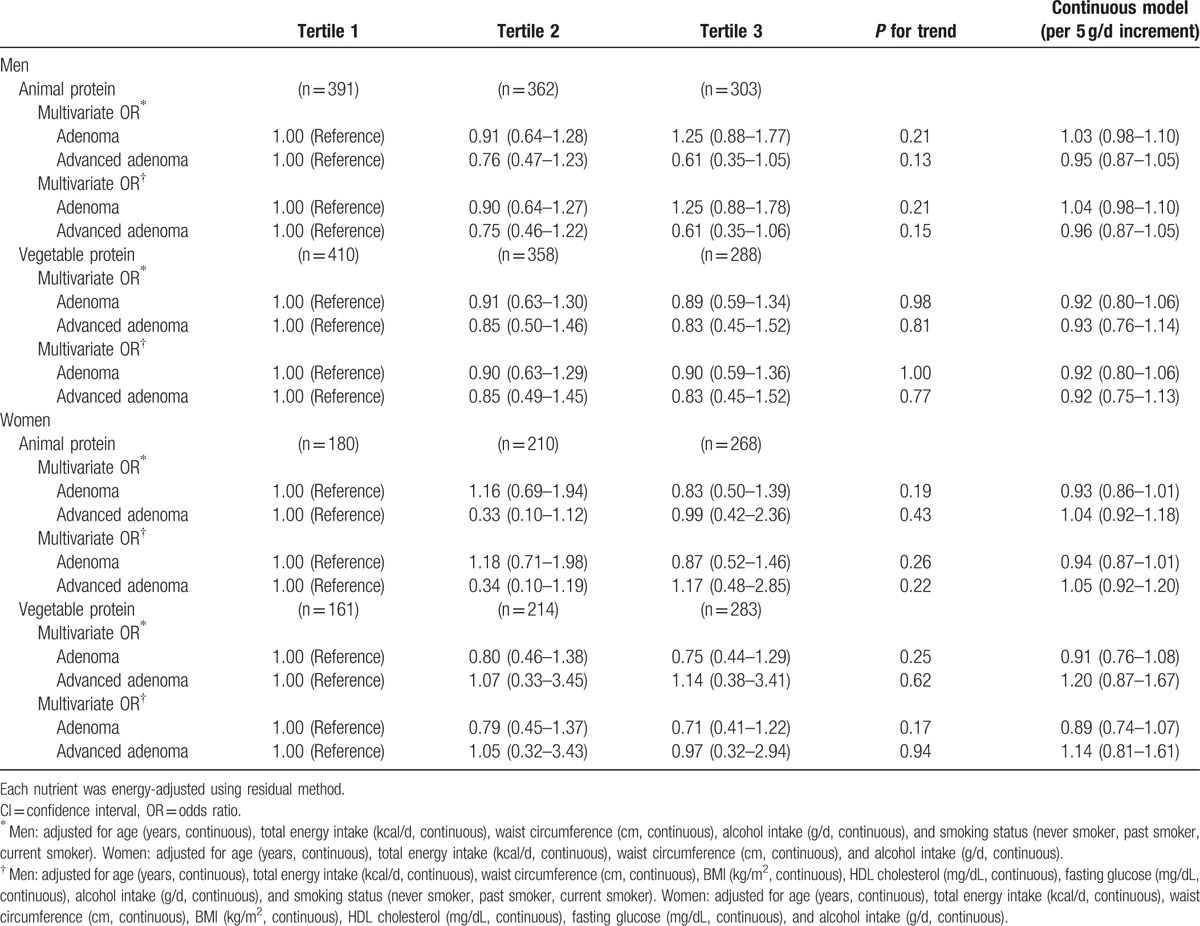
Odds ratios (ORs) and 95% confidence intervals (CIs) according to tertiles of protein intake (standard multivariate model).

**Table 3:**
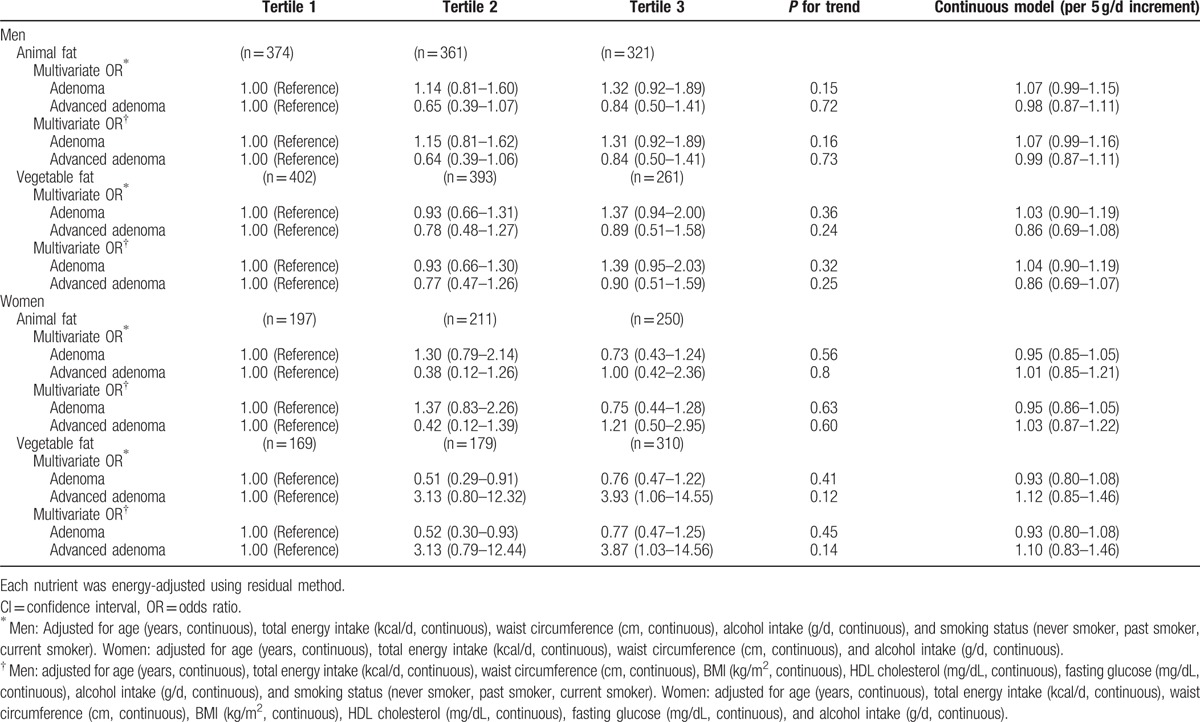
Odds ratios (ORs) and 95% confidence intervals (CIs) according to tertiles of fat intake (standard multivariate model).

When we examined the effect of replacing carbohydrate with animal and vegetable protein, and observed an inverse, but not statistically significant, association between vegetable protein intake and advanced adenoma in men after adjusting for age, total energy intake, waist circumference, BMI, HDL cholesterol, fasting glucose, alcohol intake, and smoking status (Table 4). For women, there was also an inverse, but not statistically significant, association between vegetable protein intake and adenoma or advanced adenoma. Neither animal fat intake nor vegetable fat intake was associated with the risk of colorectal adenoma or advanced adenoma when we used substitution models in which carbohydrate was replaced (Table 5).

**Table 4:**
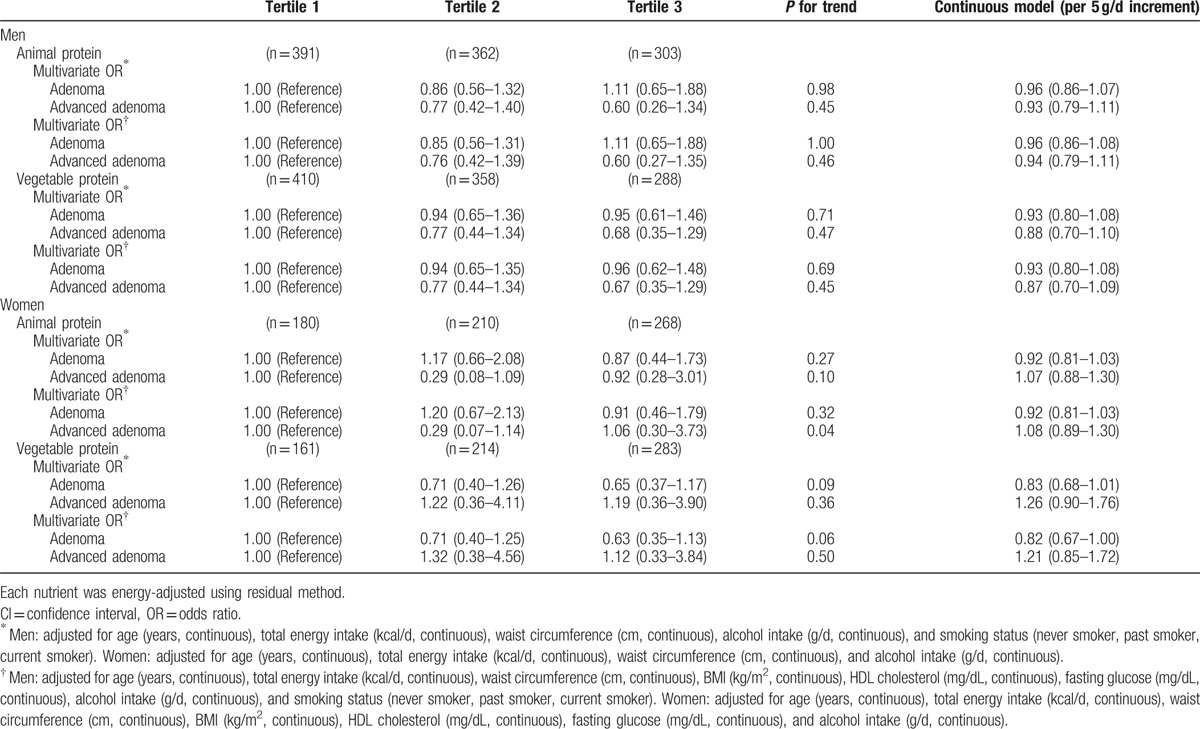
Odds ratios (ORs) and 95% confidence intervals (CIs) according to animal or vegetable protein intakes (substitution model) by replacing each for carbohydrate intake.

**Table 5:**
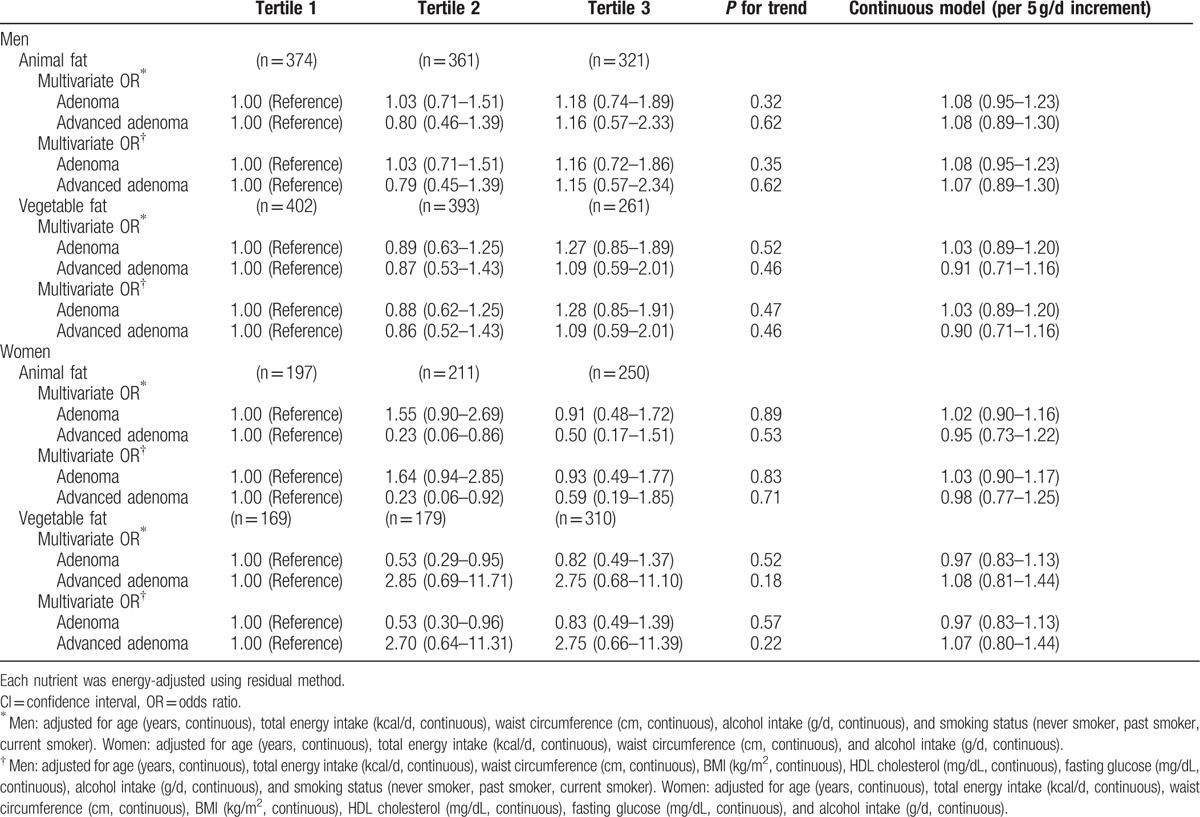
Odds ratios (ORs) and 95% confidence intervals (CIs) according to animal or vegetable fat intakes (substitution model) by replacing each for carbohydrate intake.

### 3.3 Age-matching substitution model

We also conducted a matched analysis, matched by age and sex (557 cases and 557 non-cases). For vegetable protein in women, the OR for the comparison of those in the highest tertile with those in the lowest tertile was 0.47 (95% CI 0.25–0.91, *P* for trend = 0.07) after adjustment for total energy intake (Table 6). However, after controlling for metabolic syndrome markers, BMI, smoking status, alcohol consumption, and family history of colorectal adenoma, which were all significantly high in the colorectal adenoma patients group, the association became attenuated (OR 0.54, 95% CI 0.27–1.11, *P* for trend = 0.13). There were no significant associations between dietary fat intake and colorectal adenoma in this model (Table 7).

**Table 6:**
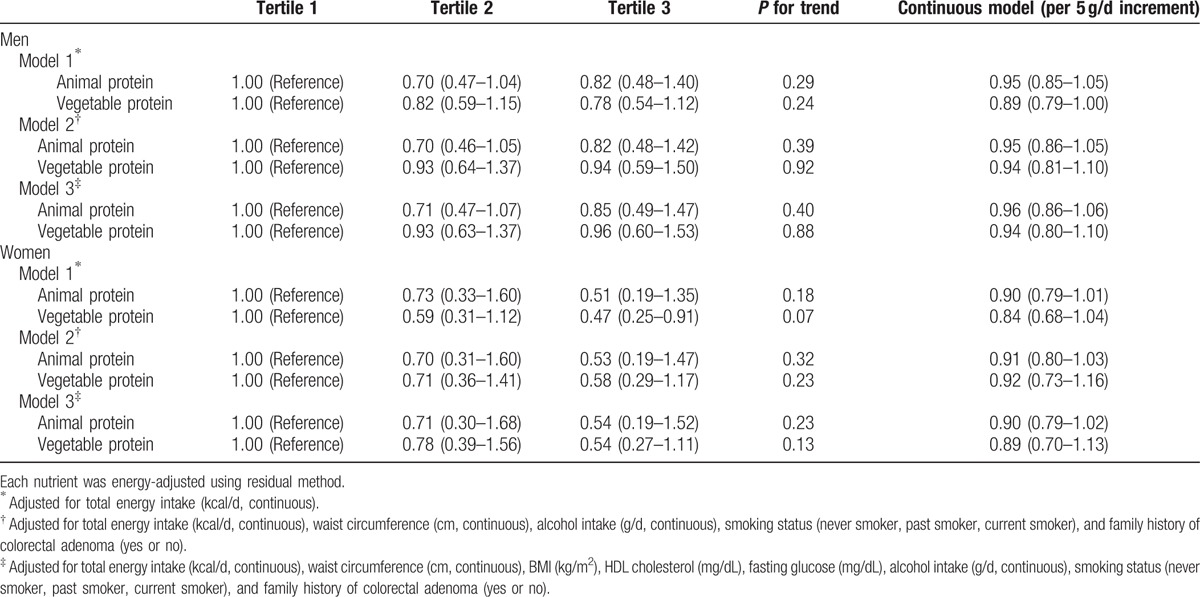
Conditional logistic odds ratios (ORs) and 95% confidence intervals (CIs) according to animal or vegetable protein intakes (substitution model) by replacing each for carbohydrate intake.

**Table 7:**
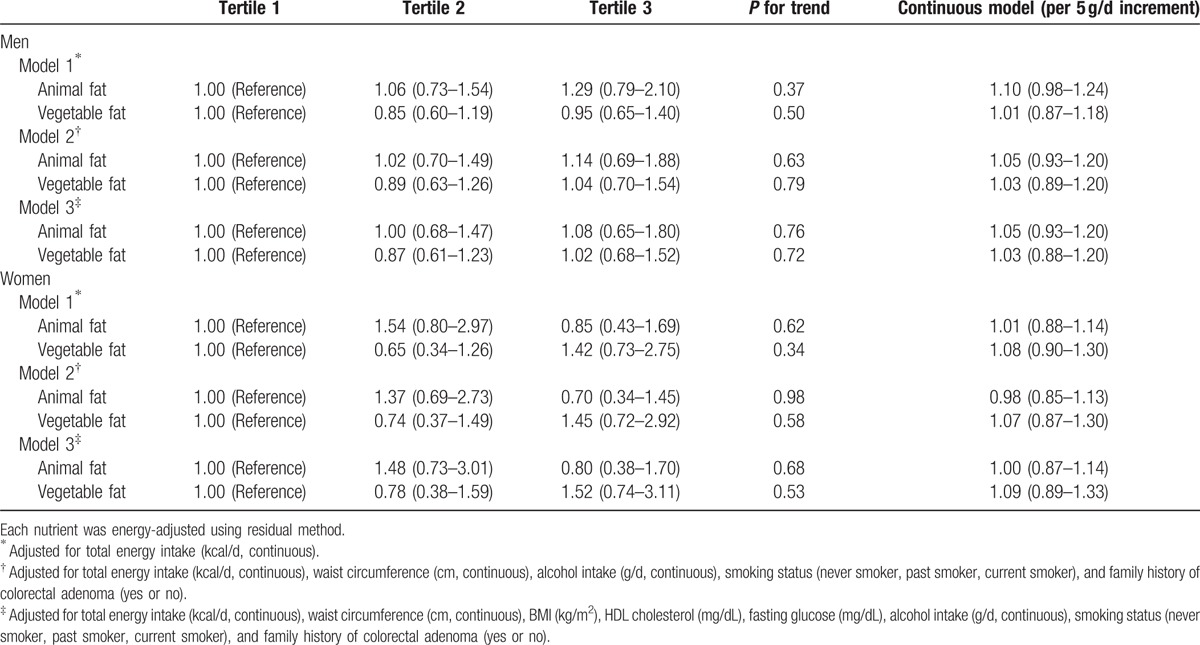
Conditional logistic odds ratios (ORs) and 95% confidence intervals (CIs) according to animal or vegetable fat intakes (substitution model) by replacing each for carbohydrate intake.

## 4 Discussion

We found that vegetable protein intake in women was inversely associated with the prevalence of colorectal adenoma in age-adjusted models; further adjustment for potential confounding factors including waist circumference, BMI, HDL cholesterol, fasting glucose, alcohol consumption, smoking status, and family history of colorectal adenoma attenuated the association, resulting in no significant associations.

Our previous study revealed that both male and female patients with colorectal adenoma showed high total energy and animal protein intakes.^[23]^ However, the study had limitations in that it was a retrospective cross-sectional case-control study, and because it was based on a 24-hour dietary recall survey, it could not be considered fully representative of daily meals. To overcome these limitations, the present study was conducted as a prospective study to examine patients who underwent colonoscopy as part of a health check-up.

Western studies have shown a positive association between the risk of colon cancer and high total energy intake,^[24–26]^ and laboratory studies have shown a reduction in colon cancer progression when patients followed a calorie-restrictive diet.^[27,28]^ In the present study, total energy intake did not show a significant difference between the patient and control groups. Unlike previous studies, the average total energy intake of both groups was lower than the previous 24-hour dietary recall survey results, and no statistically significant difference was observed between the 2 groups. This seems to be because the data for analysis were collected by using a FFQ survey, which reflects a long-term diet. As the 24-hour dietary recall survey relies on memory, it can produce an overestimation of actual intake.^[29,30]^

It is difficult to analyze how total energy intake affects the incidence of a disease. This is because a high calorie intake means that abundant nutrition is provided to the body, resulting in an abundance of beneficial nutrients that prevent diseases. In addition, other factors affect energy intake, including body size, physical activity, and metabolic rate.^[31]^ Studies on the relationship between macronutrients and diseases recommend correcting for total energy intake while determining whether the risk of a disease is associated with the intake of macronutrients, rather than total energy intake. However, correcting for total energy intake may weaken the relationship between individual macronutrients and the disease. For example, a study was conducted on the relationship of colon cancer incidence with macronutrient and total energy intakes in African American and Whites. In the study, the cancer patients and normal control groups were analyzed both with and without corrections for total energy intake. Among white individuals, when total energy intake was not corrected for, protein, carbohydrates, and fat increased the risk of colon cancer by 2.9, 2.8, and 2.8 times, respectively. However, after correction for total energy intake, no statistical significance was detected.^[32]^

In the present study, both male and female patients with colorectal adenoma showed significantly higher age, triglyceride, and fasting blood glucose level compared with non-cases. In particular, the female patients had also higher alcohol consumption and higher proportion of obesity compared with non-cases. The male and female patients both showed a higher proportion of hypertension, whereas the female patients also showed higher proportions of diabetes and hyperlipidemia. Adjustment for alcohol consumption, metabolic syndrome markers, and total energy intake, which are well-known risk factors of colorectal adenoma, weakened the association between colorectal adenoma and individual macronutrients. Although not statistically significant, an inverse association was found between the incidence of colorectal adenoma and intake of vegetable protein in the female patients. The intake of vegetable protein is known to be effective for regulating BP, cholesterol level, and body weight, and is thought to have a preventive effect against metabolic syndrome. In case of cholesterol, in a meta-analysis of 38 studies, soy protein diet was found to lower levels of total cholesterol by 9.3%, low-density lipoprotein (LDL) cholesterol by 12.9%, and triglycerides by 10.5%, and to increase HDL cholesterol by 2.4%.^[33]^ On the association between vegetable protein intake and cancer incidence, a study found that Asian and Asian American individuals who ate soy food or soy protein in childhood and adolescents showed protective effects against breast cancer. However, no studies have investigated the correlation with colorectal adenoma or cancer.^[34,35]^ For colorectal adenoma or cancer, an analysis of nutritional risk factors revealed that early life exposure was more important than diet at the time of diagnosis; therefore, further research is required in this field.

This study had some limitations. First, our study was a cross-sectional design; therefore a causal inference may not be clear. However, participants provided dietary information before colonoscopy; thus recall bias may not be likely. Second, although the subjects of the research were healthy people who underwent health examinations, they were people who chose a single health promotion center. Therefore, they cannot be assumed to represent the nutrition intake of all Koreans, which indicates a sample selection bias. In addition, as they are people who undergo health examinations regularly, they tend to be more interested in their health care than the average population and are well-educated about health care, leading to further differences in diet and exercise with an average Korean. Third, a survey on physical exercise, which is known as a strong confounding factor when evaluating nutritional risk factors, could not be properly adjusted in this study. Generally, people who are physically more active tend to eat healthier and be more interested in health; therefore, correcting for physical exercise level is important when analyzing correlations between diet and risk of disease. However, adjustment for other healthy lifestyle factors attenuated the associations toward no association. Further adjustment for physical activity would have showed still no association.

The incidence of colorectal adenoma—a precancerous lesion of colon cancer—is increasing in Korea and other Asian countries. Western studies have reported the association between dietary factors and the disease. However, it seems likely that risk factors of the incidence of colorectal adenoma will differ between various ethnic groups. Despite the aforementioned limitations, this study is important as a prospective case-control study conducted with Korean subjects to evaluate macronutrient risk factors of colorectal adenoma and advanced adenomas.

In conclusion, this study showed no clear relationship between the incidence of colorectal adenoma/advanced adenoma and protein/fat intake. In the female patients, vegetable protein intake and the incidence of the disease showed an inverse association in the age-adjusted models, but became statistically not significant in the full-adjusted models.
